# β1-6 branching of cell surface glycoproteins may contribute to uveal melanoma progression by up-regulating cell motility

**Published:** 2008-03-26

**Authors:** Małgorzata Przybyło, Ewa Pocheć, Paweł Link-Lenczowski, Anna Lityńska

**Affiliations:** Department of Glycoconjugate Biochemistry, Institute of Zoology, Jagiellonian University, Krakow, Poland

## Abstract

**Purpose:**

This study investigated the influence of integrin expression as well as the oligosaccharide structure of surface N-glycoproteins on cell behavior of two primary uveal (92–1 and Mel202) and two primary cutaneous (FM55P and IGR-39) melanoma cell lines.

**Methods:**

Cell adhesion to fibronectin and cell migration on fibronectin (wound healing) were selected as the studied cell behavior parameters. The percentage of cells positive for expression of selected integrins was estimated by flow cytometric analysis. The influence of β1–6 branched complex-type N-oligosaccharides on wound healing on fibronectin was investigated. Cell surface β1–6 branched N-oligosaccharides were measured by their specific binding to PHA-L followed by flow cytometry, and the fibronectin receptors bearing β1–6 GlcNAc branched N-linked glycans were identified. In addition, the transcript of GnT-V (the enzyme that catalyzes the addition of N-acetylglucosamine to the core mannose of di- and tri-antennary N-glycans through a β1–6 linkage) was analyzed by semiquantitative RT–PCR.

**Results:**

Unlike the two examined cutaneous melanoma cell lines, neither of the uveal melanoma cells adhered to fibronectin. The adhesion efficiency of IGR-39 cells was twice that of FM55P cells. In contrast, uveal melanoma cells repaired scratch wounds on fibronectin-coated surfaces twice as fast as cutaneous melanoma cells did. The expression of α_3_β_1_, α_4_β_1_, α_5_β_1,_ and α_v_β_3_ integrins, acting as fibronectin receptors, differed between the tested cell lines, and no distinct pattern distinguished uveal melanoma from cutaneous melanoma except for high expression of α_4_β_1_ integrin on both FM55P and IGR-39 cells. The results also demonstrated that the high levels of α_3_β_1_, α_4_β_1_, and α_5_β_1_ integrin expression on IGR-39 cells promoted their strong attachment to fibronectin-coated surfaces. In addition, 92–1, Mel202, and FM55P cells showed no or low adhesion to fibronectin, perhaps the result of low expression of fibronectin receptors excluding high expression of α_4_β_1_ integrin in FM55P cells. Cell migration was significantly decreased in three out of four PHA-L-treated cell lines, suggesting that β1–6 branched complex type N-oligosaccharides are critical for 92–1, Mel202, and FM55P cell motility. Semiquantitative RT–PCR analysis showed that the tested cells did not differ in mRNA levels of β1–6 –N-acetylglucosaminyltransferase V. However, FACS analysis showed that 92–1, Mel202 and IGR-39 cells expressed significantly higher amounts of β1–6 branched N-oligosaccharides on the cell surface than FM55P cells did. All examined α_3_, α_5_, α_v_, and β_1_ integrin subunits were shown to bear β1–6 branched N-linked glycans.

**Conclusions:**

The role of integrins and their N-glycosylation in the regulation of uveal melanoma growth and progression is largely unknown. These results reveal that cell surface complex-type N-glycans with GlcNAc β1–6 branches are important factors determining the migration of primary uveal melanoma cells on fibronectin.

## Introduction

Uveal melanoma (UM) is the most common primary intraocular tumor in the adult population. Although significant advances have been made in the ability to diagnose and treat primary tumors of UM, the mortality rates for UM have changed little during the past few decades [1,2]. Because metastatic disease can appear as late as 12 to 15 years after enucleation of the ocular melanoma [3], the disease is likely to have already disseminated at the time of diagnosis [4], either as circulating malignant cells or as occult micrometastatic lesions [5]. UMs metastasize preferentially to the liver, and the average survival period for UM is less than one year after clinical diagnosis of the lesions [2,5,6].

Little is known about the molecular mechanisms underlying the metastatic potential of UM. Although UMs and cutaneous melanomas (CM) are of similar embryological origin and as such share several common features including morphology and the properties of the melanogenesis pathway, they differ significantly in their epidemiological [7], cytogenetic [8,9], and immunological [10-12] characteristics as well as biologic behavior [13,14]. UMs almost exclusively metastasizes through the blood and preferentially to the liver, whereas CMs are capable of both lymphatic and hematogenous (often less organ-specific) spread to almost any organ in the body. Early events in the formation of metastases include the escape of tumor cells from the primary tumor site followed by invasion into the surrounding stroma [15]; these events depend on the interaction of tumor cells with the extracellular matrix (ECM). Recently we showed that primary UMs and primary CMs also differ in their adhesion to selected components of the ECM, including fibronectin (FN), laminin, and type IV collagen [16].

Successful metastasis requires an ordered series of steps, including detachment of tumor cells from the primary neoplasm, invasion into and migration through ECM, entry into blood as well as lymph vessels, transport along the circulatory system, adhesion to the endothelium, extravasation, and outgrowth in a distant organ [17,18]. Receptors that mediate cell-cell and cell-ECM adhesion have been shown to be key components in the metastatic cascade [19]. It is reported that during the progression of malignant CM, the expression profiles of several adhesion molecules undergo changes which are directly or inversely correlated with its metastatic potential [20-22]. Loss, overexpression or malfunctioning of adhesion molecules may contribute to the detachment of tumor cells from the primary tumor, local invasion and metastasis [23].

The aims of this study were (1) to compare two human primary UM cell lines (92–1, Mel202) and two human primary CM cell lines (FM55P, IGR-39) in terms of their adhesion and migration (wound healing) to FN; (2) to determine the repertoire of integrins acting as FN receptors on these melanoma cells; (3) to test whether the oligosaccharides of surface N-glycoproteins influence melanoma cell behavior; (4) to measure the surface expression of β1–6 branched N-oligosaccharides on melanoma cells; and (5) to identify the FN receptors bearing β1–6 branched N-oligosaccharides.

## Methods

### Chemicals

*Phaseolus vulgaris* agglutinin (PHA-L), PHA-L immobilized on cross-linked 4% agarose, proteinase inhibitor cocktail, protamine sulfate, penicillin-streptomycin solution, Tween 20, bovine serum albumin (BSA), and poly-L-lysine were obtained from Sigma-Aldrich (St. Louis, MO). Fetal bovine serum (FBS) was from GibcoBRL^TM^ (Paisley, UK). The polyvinylidene difluoride (PVDF) membranes were products of Millipore (Bedford, MA, USA). We purchased 4-nitroblue-tetrazolium salt and 5-bromo-4-chloro-3-indolylphosphate solution from Roche Diagnostics GmbH (Mannheim, Germany) and obtained 96-well and 24-well plates coated with FN from BD Biosciences (San Diego, CA). Antibodies used in flow cytometric analysis as well as for immunodetection are listed in Table 1 and Table 2, respectively. All remaining chemicals were of analytical grade, commercially available.

**Table 1:** Range, specificity, and supplier of monoclonal antibodies used for flow cytometry experiments.

**Immunogen**	Antibody	Manufacturer
**α_3_**	M0608 (monoclonal – clone P1B5)	DAKO
**α_4_**	MAB16983 (monoclonal – clone P1H4)	Chemicon
**α_5_**	CBL497 (monoclonal – clone SAM-1)	Chemicon
**α_v_**	MAB1976 (monoclonal – clone LM609)	Chemicon
**rabbit anti-mouse F(ab')_2_**	F313 (FITC–conjugated)	DAKO
**mouse IgG1**	X0931 (monoclonal – clone DAK-GO1)	DAKO

### Cell lines and culture condition

Included in the study were four cell lines received from the ESTDAB Melanoma Cell Bank (Tűbingen, Germany). Two cell lines were derived from a primary UM (92–1 [24] and Mel202 [11]) and two others were derived from a primary CM (FM55P [25] and IGR-39 [26]).

All cell lines were cultured in RPMI-1640 medium (GibcoBRL^TM^), supplemented with 10% heat-inactivated FBS, and penicillin-streptomycin solution. Cells were fed biweekly and grown to confluence as a monolayer in 5% CO_2_-enriched atmosphere at 37 °C in a humidified incubator, and passaged by treatment with 0.05% trypsin-EDTA solution (Sigma-Aldrich). Experiments were initiated when cells had reached subconfluence.

### Adhesion assay

Melanoma cell adhesion to FN was assessed according to the protocol previously described [16]. The reference value for 100% attachment was estimated from cells in wells coated with 500 μg/ml poly-L-lysine. All data are given as relative percentages of adhesion compared to adhesion on poly-L-lysine (taken as 100%). Values are expressed as mean ± standard deviation of three separate experiments.

**Figure 1:**
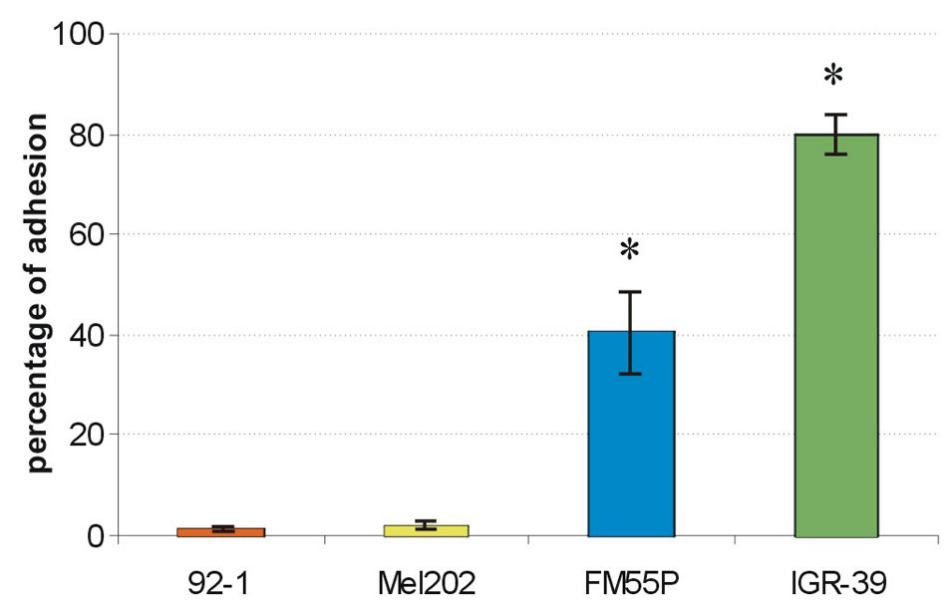
Adhesion of uveal (92–1, Mel202) and cutaneous melanoma (FM55P, IGR-39) cells to fibronectin. Each result is the average of three independent experiments done in triplicate. All data are given as percentage of adhesion relative to adhesion on poly-L-lysine (taken as 100%). Error bars indicate standard deviations. Asterisk (*) indicates p<0.05.

### Wound healing assay

Cells were grown on FN to confluence. Then the medium was aspirated, and the cell-coated surface was scraped with a 200 μl pipette tip in a single stripe. The scrape-wounded surface was washed once with PBS and twice with RPMI-1640 supplemented with FBS, and then the wounds in the cultures were allowed to heal for 24 h at 37 °C. In some experiments, wound healing in culture medium containing 25 μg/ml PHA-L was examined. The applied dose of PHA-L had no effect on the viability or growth rate of the tested cells as demonstrated by trypan blue exclusion and 3[4,5-dimethyldiazol-2-yl]-2,5diphenyltetrazolium bromide (MTT) tests (data not shown). Migration of cells into wounded areas was observed with an inverted microscope and photographed. The average extent of wound closure was quantified by multiple measurements of the width of the wound space for each of these cases. Twenty measurements of two separate trials were made and averaged for all these conditions. Values are expressed as mean ± standard deviation of three separate experiments.

### Flow cytometric analysis

Expression of human integrin subunits was assessed by flow cytometry as previously described [27]. Briefly, cells (1 × 10^5^) were incubated for 45 min on ice with antibodies against 50 μl/ml α_3_, 50 μl/ml α_4_, 75 μl/ml α_5_, integrin subunits as well as against 10 μl/ml α_v_β_3_, integrin, or 50 μl/ml normal mouse IgG as negative control, Cells were then washed in phosphate-buffered saline (PBS, pH 7.2) and then incubated with 50 μl/ml fluorescein isothiocyanate (FITC)-conjugated antimouse IgG (Fab’)_2_ fragments for 45 min on ice. PHA-L binding to cells was performed according to the method of Chakraborty et al. [28]. Briefly, cells (1 × 10^5^) were incubated with 10 μg/ml FITC-conjugated PHA-L (Vector, Burlingame, CA) in PBS containing 2% BSA, for 45 min on ice. The cells were then washed in PBS, and assessed for fluorescence in a FACSCalibur flow cytometer (BD Biosciences, San Diego, CA). A total of 10^4^ cells were analyzed for each immunofluorescence profile. Antibodies used in flow cytometric analysis are listed in Table 1.

**Figure 2:**
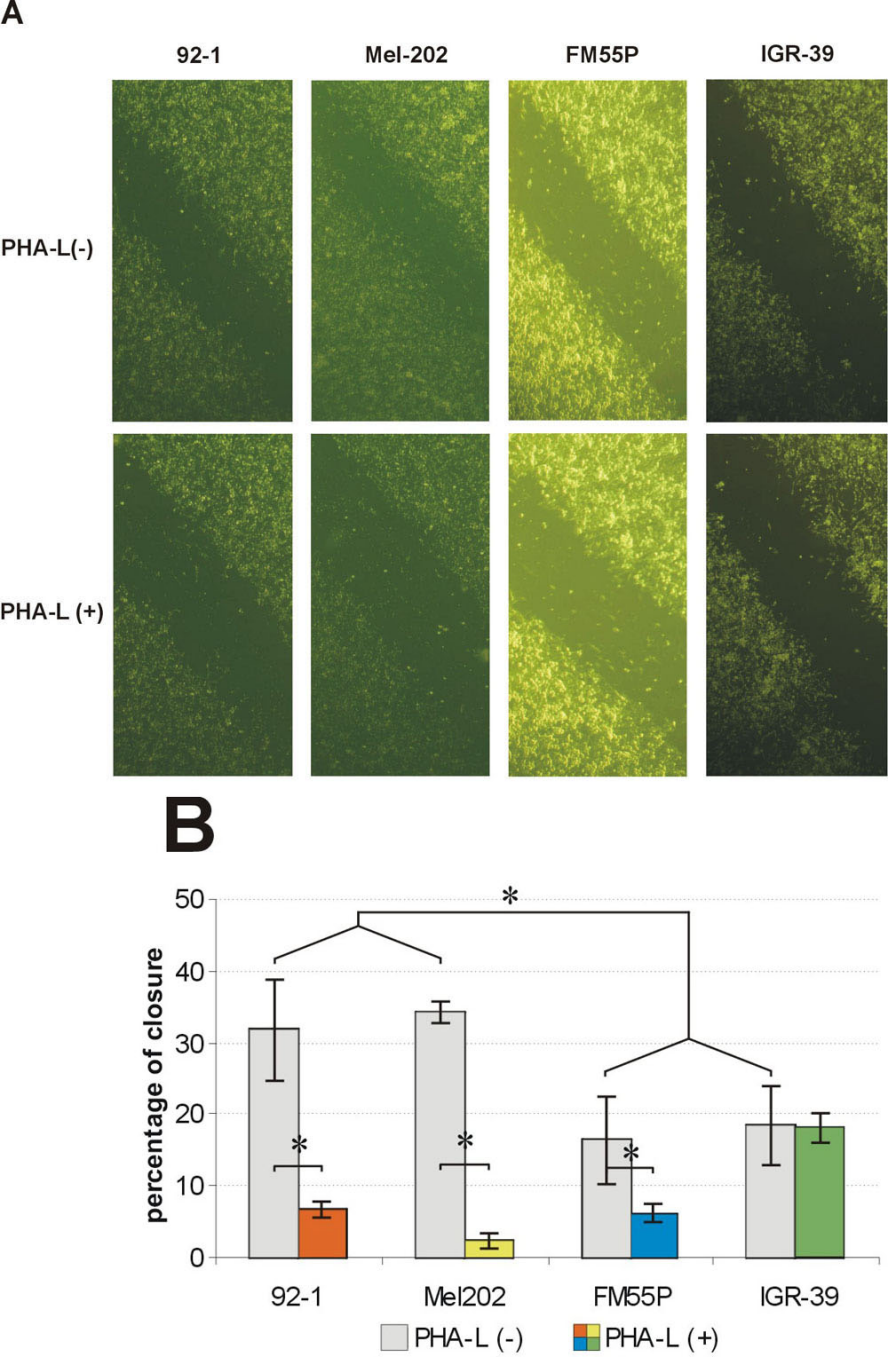
Effect of phaseolus vulgaris agglutinin on repair of wounds in monolayers of 92–1, Mel202, FM55P, and IGR-39 cells. A line was scratched with a plastic pipette tip through the confluent monolayer of cells maintained in serum-containing RPMI 1640 on a fibronectin-coated surface. The wounded cultures were allowed to heal for 24 h at 37 °C in the presence or absence of 25 μg/ml phaseolus vulgaris agglutinin (PHA-L) in serum-containing RPMI 1640. **A:** Panels show migration of cells in the presence or absence of PHAL after 24 h. **B:** The extent of wound closure was quantified by measurements of the width of the wound space for each case. For this value, the width was measured at twenty different locations in the wound and the mean value was compared to the width of the original closure (0 h). Values are means ± standard deviation of three separate experiments. Asterisk (*) indicates p<0.05.

### Expression of mRNA for β1,6-N-acetylglucosaminyltransferase V

RNA was extracted using RNeasy Mini Kit (Qiagen, Hilden, Germany). The concentration and quality of RNA samples were measured with a Spectrophotometer UV/VIS (Beckman) Then 1 μg of total cellular RNA was reverse transcribed by reverse transcriptase Omniscript (Qiagen) with oligo dT_23_ according to manufacture protocol. PCR amplification of the sample was performed with both specific primer pairs for each of the studied glycosyltransferases: β1–6-N-acetylglucosaminyltransferase V – *MGAT-5*, and human glyceraldehyde-3-phosphate dehydrogenase *(GAPDH*) genes. The PCR reaction comprised 30 cycles and consisted of denaturing at 94 °C (1 min), annealing at 60.5 °C (1 min), and extension at 72 °C (2 min). The PCR mixture contained: 2.5 μl 10x PCR buffer, 5 μl Q-solution, 1 μl MgCl_2_, 2 μl sample cDNA, 1.6 μl 10 mM dNTPs, 0.25 μl Taq DNA polymerase (Qiagen), 1 μl of each glycosyltransferase specific primer (concentration 10 μM), and water in a final volume of 22 μl. The negative control reaction was performed simultaneously. Reaction products obtained after 30 cycles were electrophoresed on 2% agarose containing ethidium bromide. Glycosyltransferase mRNA expression of each sample was determined in at least two independent experiments (separate RNA isolation) and was normalized relative to GAPDH values. Sequences of forward (F) and reverse (R) oligonucleotide primers for *MGAT-5* gene and length of the amplification products were as follows: F: 5′-GTG GAT AGC TTC TGG AAG AA-3′ R: 5′-CAG TCT TTG CAG AGA GCC-3′ (856 bp).

**Table 2:** Range, specificity, and supplier of monoclonal and polyclonal antibodies used for immunodetection of integrin chains in material recovered after precipitation with phaseolus vulgaris agglutinin bound to agarose.

**Immunogen**	Antibody	Manufacturer
**α_3_**	AB1920	Chemicon
**α_5_**	AB1928	Chemicon
**α_v_**	AB1930	Chemicon
**β_1_**	MAB2251 (monoclonal – clone B3B11)	Chemicon
**anti-rabbit IgG**	AP322A (AP–conjugated)	Chemicon
**anti-mouse IgG**	A1682 (AP-conjugated)	Sigma

### Precipitation with *Phaseolus vulgaris* agglutinin lectin

After reaching early confluency, cells were washed twice with PBS, harvested with a rubber policeman and pelleted by centrifugation. Then the cells were homogenized on ice in 10 mM Tris/HCl, pH 7.5, containing 1 mM EDTA and a proteinase inhibitor cocktail, followed by incubation with the same buffer containing additionally 1% Triton X-100 and 0.3% protamine sulfate for 1 h on ice. Finally, cell extracts were clarified by centrifugation at 16000 g for one hour.

Precipitation with the use of immobilized PHA-L lectin was performed according to the modified method of Seales et al. [29]. Briefly, 1 mg cell extracts were incubated overnight at 4 °C with 50 μl of PHA-L immobilized on cross-linked 4% beaded agarose, 3 mg lectin/ml packed gel. PHA-L/glycoprotein complexes collected by brief centrifugation were then washed three times with 10 mM HEPES, pH 7.5, containing 150 mM NaCl, followed by one wash with PBS. Glycoproteins were released from the complexes by boiling in electrophoresis sample buffer before being subjected to SDS–PAGE. Plain agarose was used as negative control (data not shown).

**Figure 3:**
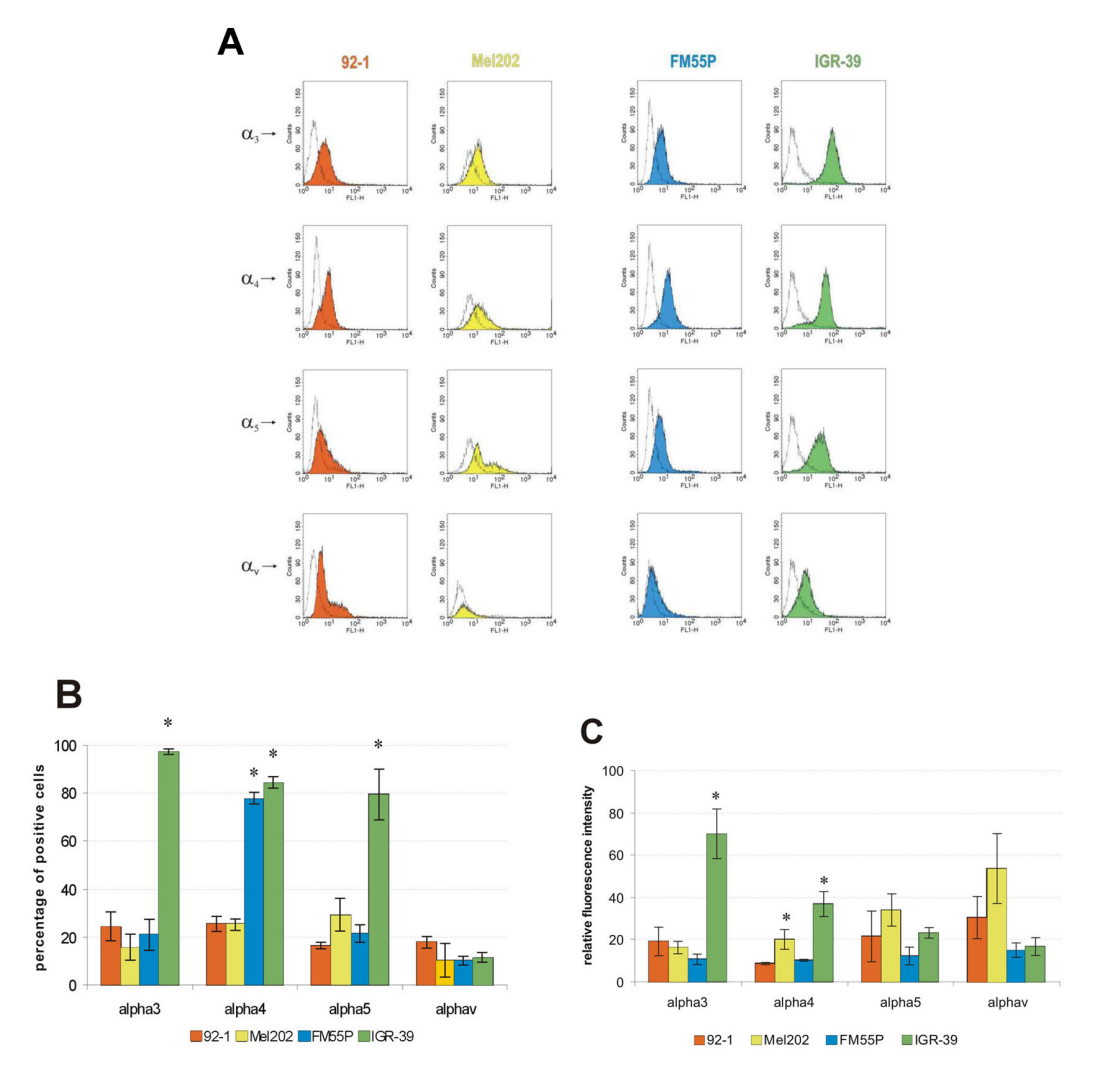
Expression of integrins on human uveal (92–1, Mel202) and cutaneous melanoma (FM55P, IGR-39) cells. Melanoma cells were examined by flow cytometry for the expression of α_3_β_1_, α_4_β_1_, α_5_β_1_, and α_v_β_3_ integrins, and data were compared to cells incubated with normal mouse IgG. Fluorescein isothiocyanate (FITC)-conjugated rabbit anti-mouse IgG (Fab’)_2_ fragments were used for detection. Fluorescence signals of 10,000 cells were counted for each integrin subunit tested. Histograms of cells versus log fluorescence were generated. **A:** Panels show FACS profile for integrin-positive cell lines. Colored areas indicate the fluorescence profile of cells after indirect fluorescence staining with anti-integrin monoclonal antibodies. Open histograms represent background fluorescence. Relative fluorescence is shown as a logarithmic scale of 4 log cycles on the x-axis, and cell number as a linear scale on the y-axis. Data from one of three similar experiments are presented. The negative control for each line is different in some experiments because the experiments were not run on the same occasion. **B:** Diagram shows percentage of melanoma cells expressing α_3_β_1_, α_4_β_1_, α_5_β_1,_ and α_v_β_3_ integrins. **C:** Diagram shows quantitation of data from flow cytometric analyses. Values are means ± standard deviation of three separate experiments. Asterisk (*) indicates p<0.05.

### SDS–PAGE and western blotting

For electrophoresis, equal volumes of proteins (1/20 of precipitated materials) mixed with sample buffer and heated, were separated on 10% SDS-polyacrylamide gels under nonreducing condition according to the method of Laemmli [30]. Following separation, the proteins were transferred onto PVDF membranes in buffer consisting of 25 mM Tris, 0.192 M glycine, and 20% methanol, pH 8.4, overnight at constant amperage 0.1 A with cooling (Bio-Rad). Polyacrylamide gels were calibrated for molecular weight determination using the Sigma Standard Kit for electrophoresis in SDS (205–29 kDa).

### Identification of the proteins bearing β1–6 GlcNAc branched N-linked oligosaccharides

Materials precipitated with PHA-L lectin were separated by SDS–PAGE and blotted onto PVDF membrane. Next the blots were blocked with 1% BSA in 20 mM Tris/HCl, pH 7.6, containing 0.15 M NaCl and 0.1% Tween 20 (TBS/Tween). Afterwards, the membranes were incubated for 2 h in 1% BSA in TBS/Tween containing a 1:1000 dilution of one of the following antibodies specific for different integrin subunits: α_3,_ α_5_, α_v_, and β_1_. Following a triple wash with TBS/Tween, the membranes were incubated for 1 h with the secondary antibodies either alkaline phosphatase conjugated goat anti-rabbit IgG (for α_3_, α_5_, α_v_, integrin subunits; 1:250 dilution in TBS/Tween with 1% BSA) or alkaline phosphatase coupled goat anti-mouse IgG (for β_1_ integrin subunit; 1:500 dilution in TBS/Tween with 1% BSA). Visualization of immunoreactive proteins was achieved with the use of 4-nitroblue-tetrazolium salt/5-bromo-4-chloro-3-indolylophosphate solution. Antibodies used for immunodetection are listed in Table 2.

### Statistics

**Figure 4:**
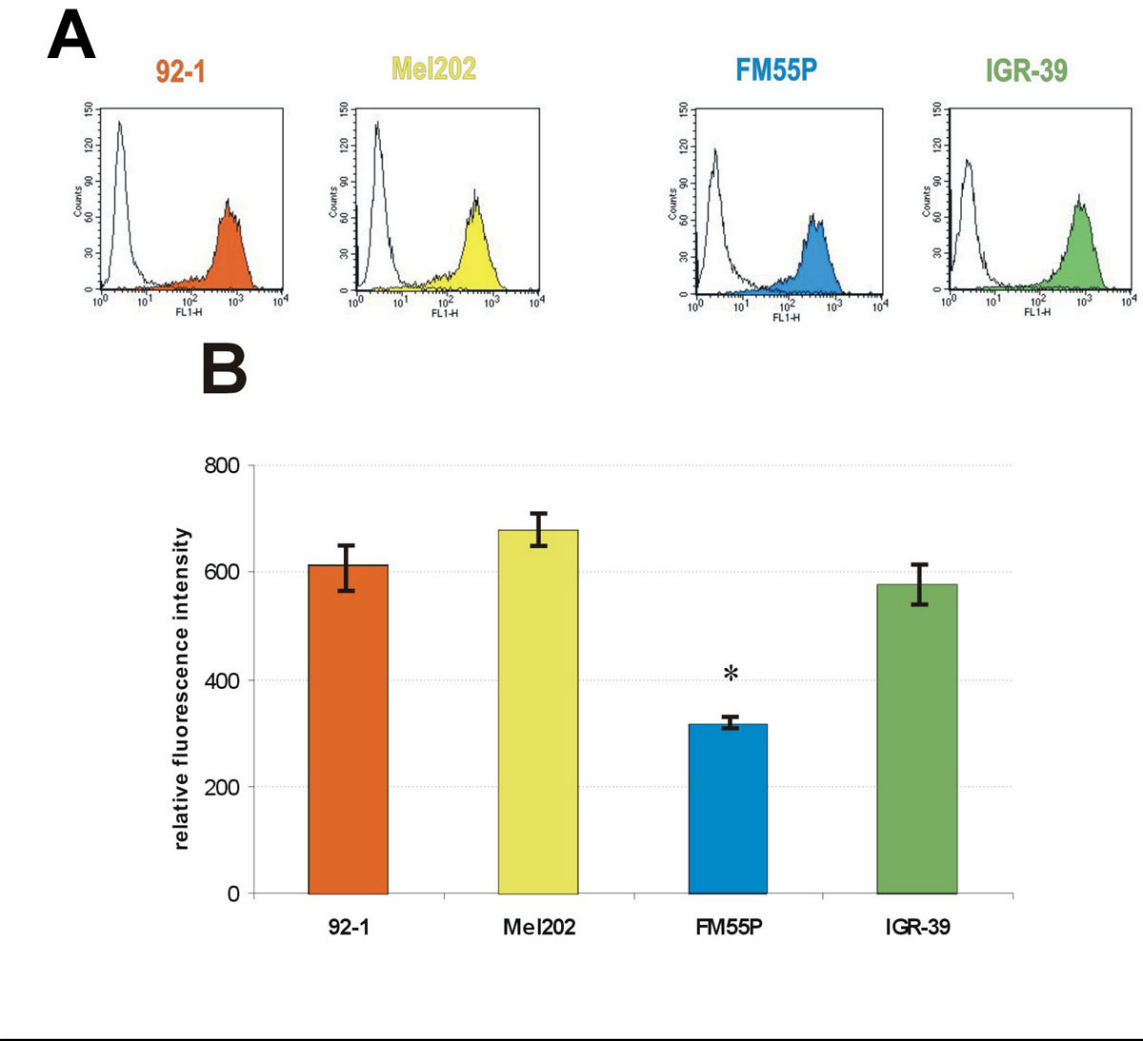
Flow cytometric analysis of phaseolus vulgaris agglutinin binding on the surface of human uveal (92–1, Mel202) and cutaneous melanoma (FM55P, IGR-39) cells. **A:** Histogram of fluorescence intensity with or without fluorescein isothiocyanate (FITC)-conjugated phaseolus vulgaris agglutinin. **B:** Quantification of data from flow cytometric analyses. Fluorescence intensity relative to negative control, representing means from three pooled experiments. Asterisk (*) indicates p<0.05.

Statistical analysis was performed with the use of Dunkan’s new multiple range test. A *p* value less than 0.05 was considered statistically significant.

### Other methods

Protein concentrations were measured according to Peterson [31] with BSA as standard.

## Results

Cell-FN interaction, mediated through several different receptors, has been implicated in a wide variety of cell activities, including important roles at several stages of tumor development [32]. In the first part of this study we compared primary UM and primary CM in terms of their adhesion and migration to FN. UM cells did not adhere to FN. As shown in Figure 1, the two examined CM cell lines attached to FN, but the adhesion efficiency of IGR-39 cells (80%) was twice that of FM55P cells (40%). Interestingly, UM cells repaired scratch wounds twice as fast as CM cells did (36% and 18% wound closure after 24 h, respectively; Figure 2).

Among the various classes of cell adhesion molecules, integrins are particularly associated with cell adhesion to extracellular matrices, and altered levels of integrin expression are related to tissue invasion and metastasis in many types of cancer. In the second part of this study we used flow cytometry to characterize UM and CM cells with respect to their cell surface integrins acting as receptors for FN (α_3_β_1_, α_4_β_1_, α_5_β_1_, α_v_β_3_), applying specific monoclonal antibodies that recognize different integrin heterodimers or integrin subunits (Table 1). The flow cytometry data are summarized in Figure 3. FN receptor expression differed between the tested cell lines, but no distinct pattern distinguished UM from CM except for high expression of α_4_β_1_ integrin on both FM55P and IGR-39 cells. The results also showed that the high levels of α_3_β_1_, α_4_β_1_ and α_5_β_1_ integrin expression on IGR-39 cells correlate with strong attachment to FN-coated surfaces, and the high expression of α_4_β_1_ integrin on FM55P probably was enough to make them adhere weakly to FN. Interestingly, the expression of α_5_β_1_ integrin, which is known to be a major FN receptor, was low on 92–1 (16%), Mel202 (29%), and FM55P (22%) cells.

Most integrins are able to bind different ligands with different affinities. The affinity of integrins may vary depending on the cell type in which they are expressed or as the result of conformational changes. Although the molecular basis of adhesion molecule-ligand interaction is not fully understood, integrin glycosylation represents a kind of regulation by which a wide variety of these receptors have their specificity and affinity modulated in several cell lines [33-38]. Because one of the common structural alterations in cell surface glycans observed in various human and rodent tumors is highly elevated expression of β1–6-N-acetylglucosamine (β1–6 GlcNAc) branched tri- and tetraantennary complex type N-glycans [39], we also tested their influence on melanoma cell migration. Addition of PHA-L, whose preferred ligands are β1–6 branched N-glycans [40], reduced the rate of 92–1, Mel202, and FM55P cell migration into scratch wounds on FN-coated wells by 79%, 93%, and 63%, respectively, indicating the participation of β1–6 branched N-oligosaccharides in this process, but it had no effect on the migration rate of IGR-39 cells (Figure 2).

The formation of β1–6 branches on the trimannosyl terminus of N-linked oligosaccharides is controlled via the activity of GnT-V, the enzyme that catalyzes the addition of N-acetylglucosamine to the core mannose of di- and triantennary N-glycans through a β1–6 linkage [41]. Analysis of the transcript of GnT-V by semiquantitative RT–PCR showed that the tested cells did not differ in their mRNA levels of GnT-V (data not shown). To assess GnT-V activity in vivo, we measured cell surface β1–6 branched N-oligosaccharides via their specific binding to PHA-L and detection by flow cytometry. It showed that 92–1, Mel202, and IGR-39 cells expressed significantly higher amounts of β1–6 branched N-oligosaccharides on the cell surface than FM55P cells did, as reflected in the mean fluorescence intensity of the cells (Figure 4): the former cells showed mean fluorescence intensity twice that of FM55P cells. Presumably the enhanced PHA-L binding was restricted to the cell surface, because the binding and wash procedures were performed on ice with no previous lysis of cells.

**Figure 5:**
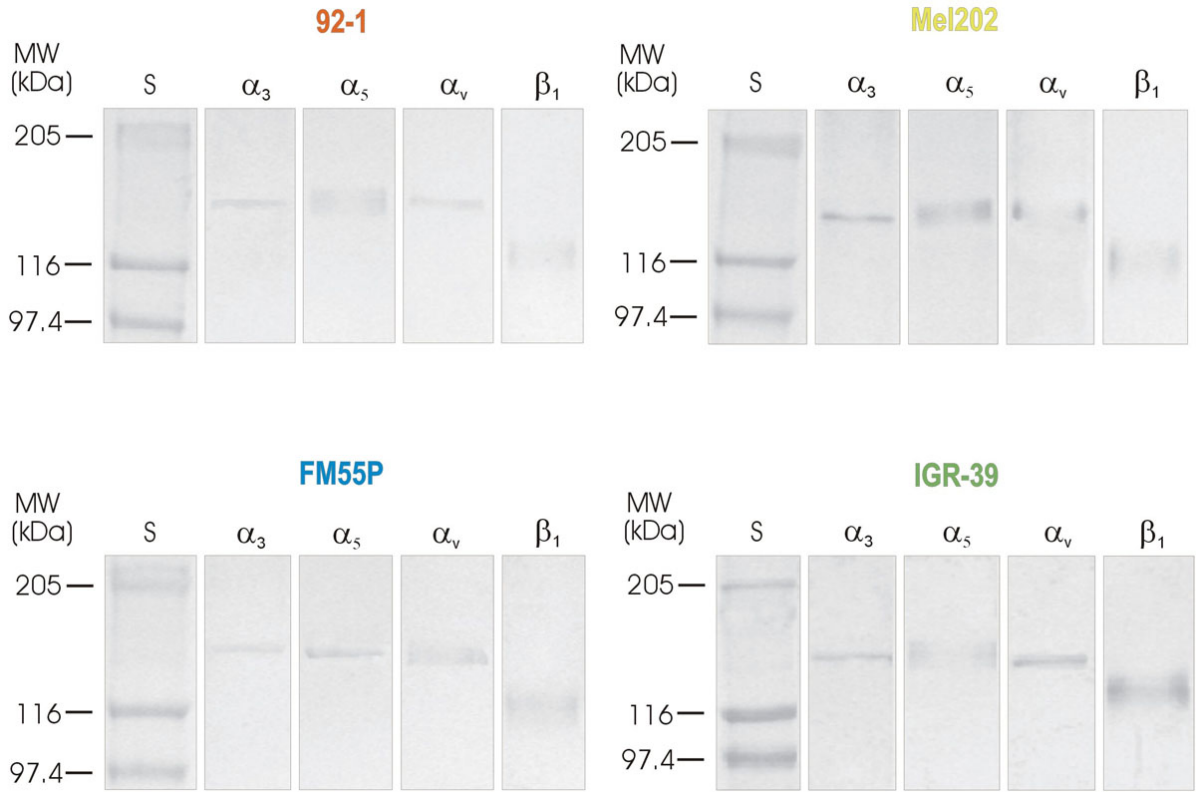
Immunodetection of α_3_, α_5_, α_v_, and β_1_ in materials obtained after precipitation of 92–1, Mel202, FM55P, and IGR-39 cell extracts with phaseolus vulgaris agglutinin bound to agarose. One mg of the cell extracts were incubated overnight with phaseolus vulgaris agglutinin (PHA-L) immobilized on cross-linked 4% beaded agarose. Glycoproteins were released from the complexes by boiling in electrophoresis sample buffer before being subjected to 10% SDS–PAGE. Following separation, the proteins were blotted onto PVDF membrane. After being blocked the blots were incubated with one of the following antibodies specific for different integrin subunits: α_3_, α_5_, α_v,_ and β_1_. Next, the membranes were incubated with the secondary antibodies either alkaline phosphatase conjugated goat anti-rabbit IgG (for α_3_, α_5_, α_v_, integrin subunits) or alkaline phosphatase coupled goat anti-mouse IgG (for β_1_ integrin subunit). Visualization of immunoreactive proteins was achieved with the use of 4-nitroblue-tetrazolium salt/5-bromo-4-chloro-3-indolylophosphate solution. Lane S shows position of molecular weight markers.

To identify the glycoproteins bearing β1–6 GlcNAc branched N-glycans from four melanoma cell lines, we precipitated clarified lysates of 92–1, Mel202, FM55P, and IGR-39 with PHA-L lectin. The glycoproteins recovered after precipitation were electrophoresed under nonreducing conditions, blotted onto a PVDF membrane, and probed with antibodies specific for different integrin subunits: α_3,_ α_5_, α_v_, and β_1_. Immunodetection clearly indicated the presence of β1–6 GlcNAc branched N-glycans on these integrin chains (Figure 5).

## Discussion

Many studies have shown tumor cells to be generally less adhesive and to deposit less ECM than their normal counterparts. The loosened matrix adhesion of tumor cells may permit them to leave their original site in the tissue. Previously we demonstrated that although both CM and UM are similarly derived from neuroectodermal tissues, they differ in their adhesion to type IV collagen, laminin and FN [16]. To study the biologic mechanisms that underlie this distinctive biologic behavior, we investigated the influence of the surface expression of the integrins that act as FN receptors, as well as the expression of β1–6 branched N-linked oligosaccharides on surface proteins, on UM (92–1, Mel202) and CM (FM55P, IGR-39) cell behavior. In this study we showed that the UM cells did not adhere to FN and that they repaired wounds twice as fast as CM cells did.

It is well documented in the literature that the expression pattern of adhesive molecules differs widely between normal and malignant tissues. The gain or loss of adhesive molecule expression on cancer cells appears to be a natural consequence of their adaptation and survival in a different environment. Integrins, which are the major class of adhesion molecules responsible for mediating cellular interaction with the ECM, seem to have an important role in various aspects of cancer development. Several studies have focused on the integrin expression of melanoma cells, but only the expression of α_2_β_1_, α_3_β_1_, α_6_β_1_ and α_v_β_3_ integrins was found to be associated with tumor progression in CMs [42-45]. No correlation between integrin expression and cell type or aggressive behavior has been has been confirmed in UM [46,47]. The present study found no distinct pattern of FN receptor expression among primary UMs and CMs, except for high expression of α_4_β_1_ integrin on both FM55P and IGR-39 cells. Contrasting with our results showing a high level of α_4_β_1_ integrin on CM cells are previous findings that α_4_β_1_ integrin expression was rare in CM [46]. The divergent outcomes may be attributable to differences in the techniques and antibodies used. The high levels of α_3_β_1_, α_4_β_1_, and α_5_β_1_ integrin expression on IGR-39 cells seemed to be associated with their strong adhesion to FN. Interestingly, 92–1, Mel202, and FM55P cells showed no or weak adhesion to FN, perhaps the result of low expression of FN receptors except for that of α_4_β_1_ integrin on FM55P cells. The high expression of α_4_β_1_ integrin on FM55P cells probably was enough to make them adhere weakly to FN. These results were further confirmed by assays of adhesion inhibition in the presence of specific anti-integrin monoclonal antibodies (data not shown).

There is increasing evidence that progression of cancer from a tumorigenic to metastatic phenotype is directly associated with an increased level of β1–6 branched N-oligosaccharides as the result of hyperactivity of GnT-V [41,48]. Perhaps the most interesting findings of this study are those related to the involvement of β1–6 branched N-linked oligosaccharides on surface glycoproteins in FM55P, 92–1 and Mel202 cell migration. Although literature data, including results obtained in our laboratory, describe the involvement of β1–6 branched N-glycans in CM cell adhesion and migration [49,50], the present study is the first to present data with respect to UM. The functional significance of increased β1–6 branching in N-glycoproteins has not been well established, but it has been associated with several hallmarks of tumor progression: decreased substrate adhesion, loss of contact inhibition, increased migration in vitro, and increased metastasis in vivo [28,37,41,48]. We recently showed that changes in the number of proteins acting as substrates for GnT-V were associated more with melanoma development and progression than with expression of cell adhesion molecules [50]. It is believed that expression of β1–6 branched N-oligosaccharides on integrins and other adhesion receptors may facilitate the turnover of cell-cell and cell-ECM contacts to enhance cell motility [41,51].

Here we provided evidence that β1–6 branched N-oligosaccharides on the cell surface of 92–1, Mel202, and FM55P cells facilitated their migration on FN. How is the effect of the sugar moieties achieved? Cell migration is influenced by the strength of transient cell-substratum attachment and depends on ligand and integrin levels, as well as integrin-ligand binding affinities [52]. Cell migration is most rapid at an intermediate ratio of cell-substratum adhesion to contractile force; then the cell can still extend lamellae and form new attachments at the cell front but break the attachment at the rear. In 92–1, Mel202, and FM55P cells, altered surface glycosylation might induce functional changes in adhesion proteins and in this way decrease the binding capacity of integrins to FN, possibly by holding the conformation in the low-affinity form to the ligand. Indeed, studies with tri- and tetraantennary minimal energy conformers indicate that the β1–6 branch is folded back to the protein structure [48], and this in turn could likely modulate the integrin conformation, and thus also integrin function (affinity). It has also been shown that β1–6 branched N-glycans may reduce the stability of the integrin receptor aggregates that maintain firm cell-substratum attachment, and thereby facilitate cell motility [37,41]. From flow cytometric studies it is known that 92–1 and Mel202 cells possess more glycoproteins bearing β1–6 branched N-oligosaccharides than do FM55P cells, so it is not surprising that 92–1 and Mel202 cells seemed more sensitive to PHA-L treatment as judged by their 79% and 93% versus 63% decrease in wound healing assays. Interestingly, IGR-39 cells, which possess an amount of β1–6 branched N-oligosaccharides on the cell surface similar to the level on UM cells, were not sensitive to PHA-L treatment in the wound healing assay. Possibly the high level of FN receptors on these cells had a stronger effect on the degree of transient cell-substratum attachment than did modulation of integrin-FN binding by β1–6 branched N-oligosaccharides. This suggestion needs to be confirmed.

Malignant cells acquire invasive potential by accumulating features including increased cell motility, secretion of proteolytic enzymes, and alteration of cell-substratum and cell-cell adhesion [53,54]. Tumor cell adhesion is a fundamental event in the formation of distant tumor metastases; during the formation of metastases, malignant cells often show decreased cell-cell and cell-ECM interaction at the primary tumor site and must establish new adhesive interactions at secondary sites. Elevated expression of PHA-L reactive oligosaccharides in carcinomas is usually associated with tumor progression and metastasis, as has been shown in breast and colon cancers [55,56] or melanoma [28,50]. Inhibition of the expression of β1–6 branched N-oligosaccharides through different strategies always results in the loss of metastatic ability. As 92–1 and Mel202 cells did not adhere to FN and were twice as mobile as CM cells, and since the presence of β1–6 branched N-oligosaccharides on their surface enhanced their motility, it is tempting to speculate that these cells may have also been more metastatic, but this requires confirmation.

One aspect of metastasis that has intrigued scientists for over a century is organ-specific metastasis. As mentioned, the metastatic behavior of UMs and CMs in the body differs greatly. It is believed that molecules on the surface of tumor cells are the principal regulators of adhesion to organ components. Indeed, most of the cell lines expressing β1–6 branched N-oligosaccharides have been shown to metastasize to either the liver or the lungs [41,57,58]. β1–6 branched N-oligosaccharides possibly influence adhesion by providing specific ligands to the lectin receptors on the target site, because the terminal substitution on these glycans influences the choice of metastatic site [59-62]. It has been demonstrated that β1–6 branched N-oligosaccharides substituted with polylacNAc possibly metastasize to the lungs, while cells expressing unsubstituted multiantennary N-oligosaccharides home in the liver which express galectin-1 [61,62]. UM cells metastasize specifically to the liver, so it would be useful to find clear evidence for the role of galectin-1 in liver-specific metastasis.

In 92–1, Mel202, and IGR-39 cells the levels of cell surface β1–6 branched N-oligosaccharides were significantly higher than in FM55P cells, but it did not appear to have resulted from differences in the mRNA expression of GnT-V in these cells as shown by semiquantitative RT–PCR. Nevertheless, it should be stressed that GnT-V activity may undergo regulation not only at expression level. Although little is known about the cellular regulators of GlcNAc transferases, there are few reports describing decrease in GnT-V activity in response to the metastasis gene *nm23-H1* and the tumor supressor gene *p16* [63], and loss of β1–6 GlcNAc branching of β_1_ integrins and concurrent dramatic reduction in migration through ECM after overexpression of 16-kDa membrane subunit of vacuolar H^+^-ATP-ase [64]. Moreover, it has been shown that the basal activity of GnT-V is also regulated by Ras/Raf-1/MEK/MAPK cascade and phosphatidylinositol-3-kinase/protein kinase B signaling pathway [65] and changes also during the cell cycle [66]. In addition, β1–6 branching is also dependent upon GnT-V having access to suitable oligosaccharide acceptors [67]. GnT-V competes for the same substrate as N-acetylglucosaminyltransferase III (GnT-III) [66]. Substitution by GnT-III effectively reduces β1–6 branching because GnT-V cannot act on such bisected precursor, resulting in lowering tumor cell metastasis [51,68-70]. Although it is well documented in the literature that β1–6 branched N-glycans contribute to cancer progression, the role of integrins with a bisecting GlcNAc cannot be neglected. It has been shown that the modification of α_5_β_1_ integrin by bisecting GlcNAc inhibited cell spreading and migration on FN, subsequently leading to the down-regulation of integrin-mediated signaling [70,71].

The present studies on UM cells showed remarkable homogeneity of their adhesion and migration properties, expression of FN receptors, level of β1–6 branched N-oligosaccharides on the cell surface, and the influence of these glycans on cell migration. The roles of integrins and their N-glycosylation in the regulation of UM growth and progression are largely unknown. To our knowledge, this study is the first to demonstrate the role of β1–6 branched N-oligosaccharides on surface glycoproteins in the migration of UM cells. Further studies of other melanomas are needed to confirm these interesting findings.
